# Is a good reputation a dangerous thing? A multimethod assessment of regulator culture and the implications for risk regulation

**DOI:** 10.1111/risa.70043

**Published:** 2025-04-24

**Authors:** Sharon Clarke, Lina Siegl, David Holman

**Affiliations:** ^1^ Alliance Manchester Business School University of Manchester Manchester UK

**Keywords:** culture assessment, multimethods, organizational reputation, regulator culture

## Abstract

Regulator culture has a significant influence on dutyholder safety in high‐risk industries, but there is currently limited research that has explored its nature and implications for effective risk regulation. Building on existing cultural theory and literature on reputational risk, we aim to address this hiatus by exploring regulator culture through an assessment of attitudes, beliefs, and norms that are shared within a UK risk regulator, and represent its underlying cultural values. We utilized an abductive case study approach, which involved multiple qualitative methodologies (comprising analysis of 68 documents, 19 interviews, nine focus groups, and seven observations), engaging both internal and external stakeholders. Based on triangulation, we developed a model and rich description of regulator culture, and addressed the following questions: What is a regulator culture for safety? What cultural values do risk regulators need for effective oversight of a high‐risk industry? Our model encapsulates the core values of regulator culture (process orientation, professionalism, and independence) that together support the regulator's reputation, which is central to its cultural identity. Our findings extend risk theory and research by advancing knowledge of the mechanisms through which culture impacts regulatory practice. We highlight how the drive to protect reputation has benefits, but also a potential “dark side.” Latterly, we emphasize the dynamic and paradoxical nature of cultural values, and how this affects the regulator's ability to continually improve and strengthen its culture over time, as well as the implications for effective regulatory oversight.

## 1 INTRODUCTION

Risk regulators are concerned with regulating the safety‐related performance of organizations (i.e., dutyholders) within high‐risk sectors (Baldwin et al., 2012), such as energy, chemicals, nuclear, transportation, aviation, healthcare, and the environment. Regulatory bodies take an oversight role, in which they are not directly responsible for safety (which remains the responsibility of dutyholders), but where the regulatory context can be understood as a wider institutional field, with multiple external stakeholders, including, government, NGOs, and the general public (Antonsen et al., 2017). Furthermore, risk regulators operate within differing regulatory regimes; for example, the United Kingdom encourages a largely goal‐based approach, in contrast to other countries (such the United States) which tend to be more prescriptive, or rules‐based, in nature (Decker, 2018). These contextual factors will influence the way in which risk regulators operate, including their interactions with dutyholders, and their organizational culture.

Regulator culture has been increasingly highlighted as a topic for risk and safety scientists, given its contribution to safety‐related failures, including Boeing 737 MAX crashes (House Committee, 2020), the Fukushima nuclear disaster (IAEA, 2015), and the Grenfell tower fire (Moore‐Bick, 2024). For example, the Fukushima Nuclear Accident Independent Investigation Commission (2012) concluded: “The regulatory bodies lacked an organizational culture that prioritized public safety over their own institutional wellbeing, and the correct mindset necessary for governance and oversight” (National Diet of Japan, p. 44). Furthermore, recognition of the wider environment in which high‐risk industries are located, including regulatory context, is important for situating risk and safety theory. For example, research has shown that regulator culture has a significant influence on dutyholder safety, depending on the nature of the regulator–regulatee relationship (Bernard, 2014; Willis et al., 2024).

The literature on regulator culture tends to draw on safety culture models that emphasize the internalized values and practices that are important for regulatory effectiveness such as leadership, accountability, and communication (Bernard, 2018; Bradley, 2017; Fleming & Bowers, 2016, 2019; Fleming et al., 2022). There is also another literature on regulatory effectiveness highlighting the importance of regulator reputation, that is, “a set of symbolic beliefs held by audience networks as to the actual performance of an organisation, as well as its capacities, roles, and obligations to accomplish its primary organisational mission” (Maor & Sulitzeanu‐Kenan, 2013, p. 35). This perspective emphasizes the external‐facing and political nature of regulatory bodies (Boon et al., 2023; Rimkutė, 2018) and has demonstrated how regulatory effectiveness is influenced by the way regulatory audiences perceive the regulator's reputation in successfully carrying out its role (Maor, 2015). An implication of this research is that regulator reputation is likely to be a core concern for regulators. However, the issue of regulator reputation is largely absent from models of regulator culture, which tend to be internally focused, suggesting that our theoretical and empirical understanding of regulator culture is fragmented and incomplete. Indeed, what is not clear is how regulator reputation is viewed and expressed as part of regulator culture, how external and internal‐focused aspects of regulator culture are related, and the implications of this for regulatory effectiveness.

Our aim in this paper is to develop our theoretical and empirical understanding of regulator culture by drawing on safety culture and reputation perspectives to provide a lens with which to analyze and interpret data drawn from a multimethod culture assessment (Bernard, 2021; Reiman & Norros, 2002; Reiman et al., 2005; Schöbel et al., 2017). We utilize an abductive case study approach (Dubois & Gadde, 2002), in which we focus on the case of a UK risk regulator, responsible for regulating a high‐risk industry within a goal‐based regulatory context. We start from an initial broad theoretical framework (here we draw on Schein's (1990) organizational culture theory), but through use of the methodological strategies of grounded theory (Glaser & Strauss, 1967; Strauss & Corbin, 1998), we focus on theory development by iteratively contrasting our theoretical assumptions with the reality of our grounded data (Dubois & Gadde, 2002). Schein's (1990) conceptualization identifies three levels of culture: observable artifacts (i.e., manifestations of culture that are overt and tangible), cultural values (i.e., beliefs, attitudes, and norms regarding what is prioritized), and basic assumptions (i.e., taken‐for‐granted, underlying, and largely unconscious beliefs). Culture is defined as “a pattern of basic assumptions, invented, discovered, or developed by a given group, as it learns to cope with its problems of external adaptation and internal integration, that has worked well enough to be considered valid and, therefore is to be taught to new members as the correct way to perceive, think, and feel in relation to those problems” (Schein, 1990, p. 111). Our abductive case study approach allows us to identify underlying cultural values, providing detailed description of regulator culture “as is” (Alvesson & Kärreman, 2011; Mantere & Ketoviki, 2013). It enables underlying assumptions and beliefs to emerge that are reflective of both internal and external aspects of regulator culture, while taking established cultural frameworks into account. We utilize triangulation as a means of combining data sources and methods to identify consistencies in our data, and also discrepancies, through which new interpretations and insights can emerge (Denzin, 1978; Merriam & Tisdell, 2016; Patton, 1999). From this, we develop a reputation‐focused model of regulator culture. We argue that the model helps to explain how internal cultural values and behaviors are shaped by the need to maintain a good external reputation, and how this can have positive, but also negative, implications for those cultural values and behaviors, and hence for regulatory effectiveness.

Our paper makes three main contributions to our understanding of regulator culture. First, we develop a more comprehensive and integrated theoretical account of regulator culture that provides a conceptual bridge between the literatures on safety culture and regulator reputation. Second, we explore the relationship between regulator culture and regulatory effectiveness. There is evidence that strong regulator cultures support effective regulatory practices^1^ (Bradley, 2017), but more detailed insights into this relationship are currently missing. Thus, our study contributes to a deeper understanding of *how* both internal and externally facing aspects of regulator culture facilitate effective risk regulation. Third, our study has practical value, as we make suggestions for the design and implementation of cultural assessments, and how insights can be used to strengthen cultures for effective regulation.

### 1.1 The role of culture in risk regulation

Much of the extant research on regulator culture has adopted a safety culture lens, drawing on models of safety culture in operating companies within high‐risk industries, which are directly responsible for safety performance (e.g., Bernard, 2018). Safety culture has been defined as the shared underlying beliefs, attitudes, and values that employees hold about risk and safety (Mearns et al., 1998). It is a relatively stable, multidimensional, holistic construct shared by organizational members (Guldenmund, 2000). Fleming and Bowers (2016) define “regulator safety culture” as “the product of individual and group values, attitudes, competencies and patterns of behaviour that determine the commitment to, and the style and proficiency of their approach to the regulation of industry safety” (p. 92). Existing research has focused on identifying characteristics of regulator safety culture (Fleming & Bowers, 2016, 2019; Fleming et al., 2022). While there are some commonalities with industry safety culture (such as leadership, accountability, and communication), researchers have highlighted elements unique to regulators, such as independence, systematic approach, and moral courage (Fleming & Bowers, 2016, 2019). Fleming et al. (2022) supported a five‐factor model (comprising: psychological safety, learning, collaboration and communication, accountability and responsibility, and systematic approach), where the demonstration of these characteristics leads to a strong regulator safety culture. However, there is currently limited understanding of the mechanisms involved, with explanations generally more focused on content than process. One exception is the model, developed by Vogus et al. (2010) in the healthcare sector, and later applied across industries (Bisbey et al., 2021), which focuses on interrelated actions and practices, linking cultural values to safety outcomes. These are defined as: *enabling* (i.e., leader actions that direct attention to safety and make it safe to speak up); *enacting* (i.e., front line actions that highlight threats to safety and mobilize resources to reduce them); and, *elaborating* (i.e., learning practices that rigorously reflect and feedback on safety). Thus, this model might provide guidance in exploring *how* regulator culture facilitates effective risk regulation.

Researchers have also viewed regulator culture through the lens of oversight, which emphasizes the regulatory role, and the achievement of the mission. The term “oversight culture” is preferred by some risk regulators, such as the Swiss Federal Nuclear Safety Inspectorate: “aspects of the organisational culture of the supervisory authority that relate to the exercise of its core mission” (ENSI, 2015, p. 10). Bradley (2017) found that regulatory oversight culture incorporates unique dimensions of “risk consciousness” and “systems thinking,” which do not overlap with safety culture models. Adopting an oversight lens places greater emphasis on the interactions and symbiotic relationships that the regulator has with other institutions. This is an important element, given that the way in which regulators have managed their relationships with industry, government, and other stakeholders has often emerged as a key point of regulatory failure (e.g., Vaughan, 1990). For example, the US Federal Aviation Administration's (FAA) response to pressure from the aviation industry was identified to be “at the heart of the organization's core safety culture challenges” (Final Committee Report, 2020, p. 234). Regulators must remain independent from external stakeholders, such as government and NGOs, as developing too close a relationship can affect their decision‐making (e.g., Reason, 1997; Vaughan, 1990). The oversight lens further emphasizes how culture may cross boundaries within an institutional network of stakeholders. Almklov et al. (2018) argue that taking this boundary‐spanning perspective is particularly relevant for societal safety, which is a primary concern for regulators. In a complementary stream of research, reputational scholars have emphasized how perceptions of external audiences act as a shaping force around regulatory actions and attitudes (Carpenter, 2010; Maor & Sulitzeanu‐Kenan, 2013). Researchers have highlighted how regulatory bodies seek to develop their external reputation (e.g., as credible and legitimate) by closely attending to external stakeholder perceptions (e.g., Boon et al., 2023; Rimkutė, 2018). This is important for regulators because fostering a good reputation may bring benefits, including public support, and increased autonomy (Carpenter, 2010). For example, Bianculli et al. (2017) discuss how the Spanish nuclear regulator renewed the license of the Garoña nuclear power plant, against the Spanish government's expectations of closing it down. This demonstration of independent action led to strengthening the regulator's standing (Bianculli et al., 2017). In developing our own approach, we aim to integrate these two contrasting, but complementary, lenses, gathering input from both internal and external stakeholders, to conduct our assessment of regulator culture.

### 1.2 Cultural values for effective risk regulation

Despite scholarly interest in regulator culture, there are few published cultural assessments of risk regulators. One exception is a case study of the Finnish Radiation and Nuclear Safety Authority, where, based on a multimethod approach, Reiman and Norros (2002) found that the regulator was a hierarchy‐oriented organization that emphasized control and internal processes. The core value was “effectiveness,” which underpinned other values of proficiency, firmness, impartiality, openness and cooperation, and self‐criticism. The regulator had varying responsibilities across three main roles: public (leading to credibility), expert (leading to competence), and authority (leading to effectiveness). Reiman and Norros (2002) argued that for a good regulator culture, all three roles must be in balance and organizational support structures (e.g., human resources) must be functioning well. The case study highlights both the contrasting requirements of risk regulators (i.e., public, expert, and authority roles), as well as the need to consider the organization as a whole (including corporate functions).

Although research specifically related to regulator culture is quite limited, there is a wider literature on risk regulation, which has a long history. This body of work highlights how values (such as independence) play an underpinning role in supporting effective risk regulation (e.g., Reason, 1997; Rothstein, 2003; Vaughan, 1990). Vaughan (1990) identified how interdependence between NASA and its regulatory bodies contributed to the Challenger Space Shuttle disaster in 1986, while Reason (1997) highlighted dysfunctional relationships between the regulator and regulatee (e.g., the “cosy” relationship between the UK Railway Inspectorate, and London Underground, which contributed to the King's Cross underground station fire in 1987). In a related line of research, previous work has emphasized the need for trust in regulators by external stakeholders, including both general public (Smith, 2015; Walls et al., 2004), and dutyholders (Wiig & Tharaldsen, 2012). Aligned with Reiman and Norros’ (2002) findings, earlier work identified how perceived credibility (based on organizational standing, accountability, and technical competence) underpins perceptions of the regulator as trustworthy (e.g., Frewer et al., 1996; Poortinga & Pidgeon, 2003). Thus, while not directly addressing the idea of regulator culture, this extant research highlights the type of cultural values likely to support effective regulation. The focus on values associated with credibility, competence, and trustworthiness, aligns with previous work on external reputation (e.g., Boon et al., 2023; Rimkutė, 2018) and regulator independence (e.g., Reason, 1997; Vaughan, 1990).

### 1.3 Current study

Against this backdrop, we take a holistic approach to understanding regulator culture, which integrates both safety culture and boundary‐spanning oversight lenses, and widens our enquiry across the whole organization. We draw on the literature relating to safety culture, alongside complementary work on reputational risk, recognizing that external reputation may be particularly relevant for regulators that operate in goal‐based regulatory regimes (such as the United Kingdom), where regulators depend on their powers of influence, as well as their legal authority, to encourage compliance. As a theoretical starting point for our analysis, we adopt Schein's (1990) culture model, but also utilize the process‐focused terminology developed by Vogus et al. (2010) as we progress iteratively through the analysis. We include input from external, as well as internal, stakeholders to locate the regulator within its wider institutional network (Antonsen et al., 2017). Using a triangulation approach across multiple data sources and methods (Denzin, 1978; Merriam & Tisdell, 2016; Patton, 1999), we assess organizational culture in case research of a UK risk regulator, and address the following research questions:

RQ1. What is a regulator culture for safety?
RQ2. What cultural values do risk regulators need for effective oversight of a high‐risk industry?

## 2 METHOD

**FIGURE 1:**
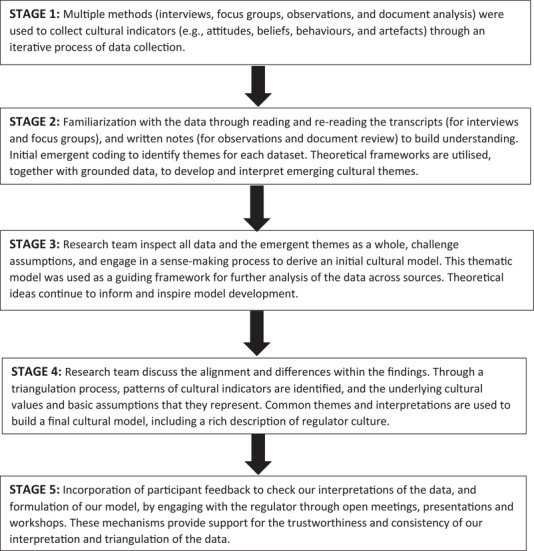
Qualitative approach to data collection, analysis, and triangulation.

Following an invitation from the regulator to conduct an independent culture assessment, we began with an initial stage of familiarization (February 2022‐March 2022), and collected data over an 8‐month period (April 2022–November 2022). Our methodological approach (which is illustrated in Figure 1) is an abductive case study that involved data collection from multiple sources (e.g., the regulator, dutyholders, and government), using multiple qualitative methods (e.g., interviews, focus groups, and observations), and triangulation across both sources and methods^2^ (Creswell & Plano Clark, 2017; Denzin, 1978; Merriam & Tisdell, 2016; Patton, 1999). Our purposeful sampling strategy aimed for maximum variation across a range of perspectives (Creswell & Plano Clark, 2017; Patton, 2002). We used triangulation to increase credibility by comparing and contrasting across sources and methods, allowing us to identify both unique differences as well as common patterns and themes, which we discussed to understand the implications.

As advocated by Casey et al. (2022), our measures aligned with the theory underpinning the methodology to gain insights into different cultural “layers” (Schein, 1990): beliefs and assumptions (e.g., interviews); values and priorities (e.g., focus groups); and artifacts (e.g., document review). We followed an iterative sequential model for data collection, in which each stage was used to inform the next. Although overlapping to some extent, we followed these stages: document review, focus groups, interviews, and observations. The familiarization stage, and the document review, allowed us to develop an understanding of the organization, and its context, and helped to inform the focus group interviews, and elite interviews conducted with senior staff, Board members, and external stakeholders (Natow, 2020). At each stage, we gained ethical approval from the University of Manchester Ethics Committee (UREC: 2022‐15121‐24769; 2022‐15362‐25407). We obtained informed consent of all participants, and ensured anonymity and confidentiality of those taking part. This included recording limited demographics, so that our participants could not be identified based on this information. In this regard, we do not report the age, gender identity, race, or sexual orientation of our participants.

We utilized a process of open (initial), axial (focused), and selective (theoretical) coding, where codes were gradually further removed from responses toward broad analytical themes (Charmaz, 2008; Strauss & Corbin, 1998). We began with an initial process of open coding in which data (e.g., interview transcripts, observation field notes) were systematically broken down into smaller segments, each assigned a descriptive label. This was followed by more focused axial coding that was used to identify relationships between the emerging themes and organize them into higher‐order conceptual categories. Finally, we used selective (or theoretical) coding at the final stage of synthesis to identify theoretically meaningful overarching themes (which in our study related to aspects of regulator culture, such as core values) (Bell et al., 2022). This was an iterative process, which allowed us to seek the best possible explanations in the context of existing theory and research (Alvesson & Kärreman, 2011; Mantere & Ketoviki, 2013).

We now describe each of the constituent data collection methods in turn.

### 2.1 Document analysis

We conducted a qualitative document analysis (Bowen, 2009), including historical background and context. We identified 68 documents, including policies, meeting minutes, internal reports on complaints/near misses/incidents, exit interviews, performance reviews, and training. Each document was analyzed for cultural indicators (e.g., artifacts, which are overt manifestations of culture, such as document wording and emphasis). We used a reflexive process of note‐taking, where the researcher made notes on each document, and discussed these with the research team to check their interpretation.

### 2.2 Focus groups

We conducted in‐person and remote focus group interviews with staff. We used a purposeful sampling approach (Creswell & Plano Clark, 2017; Patton, 2002), utilizing staff lists to invite participants from different levels (e.g., apprentices, junior and senior grades) and functional areas (e.g., inspectors, corporate functions) of the organization. In‐person groups were held at the regulator's offices, and remote groups via MS Teams. We held nine focus groups, with groups of five to six participants (50 staff in total). Each group lasted 2.5 h on average. The focus groups were audio‐recorded, and transcribed, for analysis. See the Appendix (in the Supporting Information) for the interview guide used.

### 2.3 Interviews

We held 19 interviews, including 12 members of the senior leadership team, executive and nonexecutive members of the Board, and seven external stakeholders. Participants were identified based on their professional roles (including senior inspectors who held management positions at the regulator). External participants worked for organizations that interacted regularly with the regulator (e.g., dutyholders, government departments, or other regulators). The interviews were semistructured, with questions designed to draw out participants’ understanding and experience of regulator culture, and lasted approximately 45 min on average (see the Appendix, in the Supporting Information, for the interview schedule). Interviews were conducted via MS Teams, audio‐recorded, and transcribed, for analysis.

### 2.4 Observations

We conducted seven observations including internal meetings (involving senior management and Board members) conducted as part of the regulator's normal business. We observed regulatory meetings with dutyholders and other external stakeholders (e.g., public forum involving representatives of the regulator and relevant NGOs).

Observations were conducted via MS Teams, or in‐person onsite (e.g., at the dutyholder's premises). Observers did not participate actively in the event, but were able to ask questions for clarification. Each observation was approximately 3 h on average. The purpose of each observation was to identify cultural indicators (e.g., artifacts, which are overt manifestations of culture) and record these in field notes (see the Appendix for observation guidance and prompts).

### 2.5 Triangulation and model development

We adopted a triangulation process across data sources and methods (Creswell & Plano Clark, 2017; Denzin, 1978; Merriam & Tisdell, 2016; Patton, 1999) (see Figure 1). Anonymized transcripts and field notes were analyzed (with the assistance of NVivo software), using thematic analysis (Braun & Clarke, 2006) to identify major themes and subthemes within the data (see Figure 2). We analyzed each data set separately, and then synthesized the data to identify consistencies and discrepancies (Morse, 2009). To synthesize the data, we conducted two x 3‐h workshops (with all coauthors) to compare the themes across methods and sources, discuss the findings, and resolve any differences in interpretation. As a research team, we engaged in sense making of the data, and integrated the findings into a cultural model and rich description.

FIGURE 2Data coding and themes (example based on interviews and focus groups).

**Figure:**
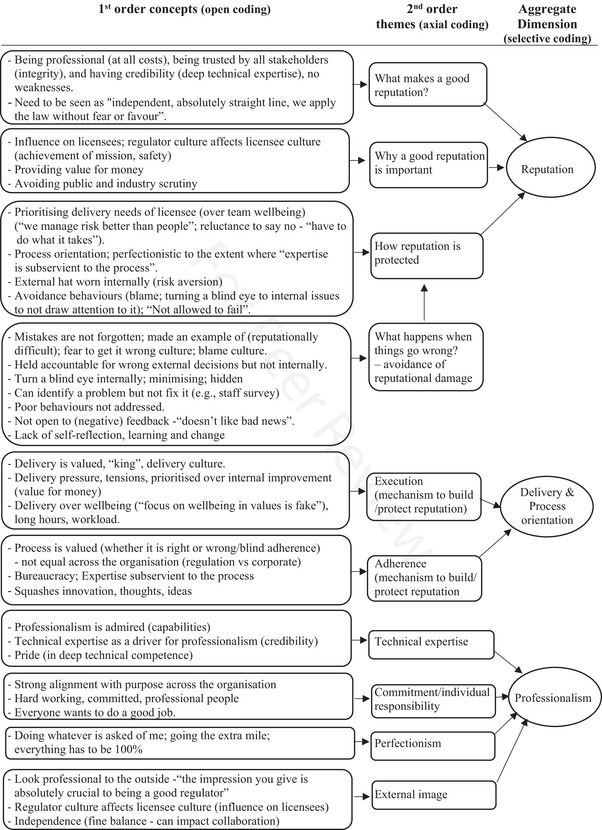

**Figure:**
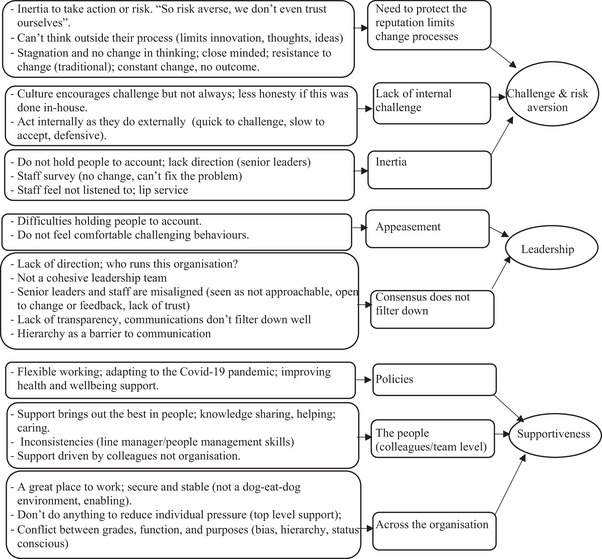

### 2.6 Participant feedback

In order to enhance the credibility (i.e., trustworthiness and consistency) of our findings, we incorporated mechanisms for participant feedback (Creswell, 2009; Loh, 2013). We checked our data interpretations, and model formulation, by engaging with the regulator, over an 8‐month period (December 2022–July 2023) at several levels (Motulsky, 2021). These mechanisms comprised: (1) feedback meeting with a small number of experienced regulatory staff, with expertise in organizational culture; (2) feedback meeting with the senior management team; (3) presentation to a Board meeting, with opportunities for questions; (4) workshop for team leaders, incorporating discussion; and (5) four well‐attended sessions open to all staff, which incorporated methods to gauge feedback. For example, one meeting used the electronic polling tool Menti to capture feedback, where staff suggested and voted on descriptors of the model. The descriptors “accurate” and “fair” were suggested and well‐supported by the staff poll.

## 3 FINDINGS

**FIGURE 3:**
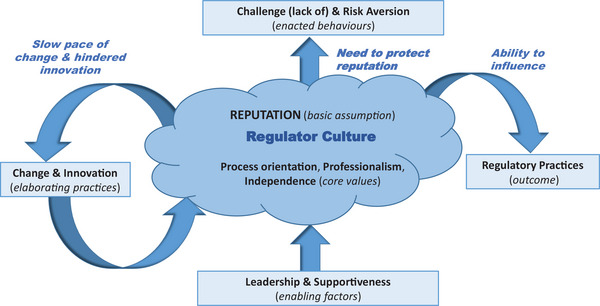
Reputation‐focused model of regulator culture.

Based on the data analysis and triangulation process, we identified patterns of attitudes, beliefs, and behavioral norms, which we integrated into a model, and accompanying rich description, of regulator culture (see Figure 3 and Table 1). Figure 3 illustrates the cultural values, attitudes, and behaviors that together comprise the regulator culture. The rich description that captures the relationships between these elements is presented in Table 1.

**TABLE 1:** Regulator culture: Rich description.

	Rich description of regulator culture
1.	At the core of the regulator's culture (as a basic assumption) was a concern with its reputation, and the central importance of being seen as an effective regulator. In large part, this was because a strong reputation was required to influence dutyholders’ behavior, which is a critical process in fulfilling the regulator's mission. Reputation was maintained through strongly held cultural values for effective delivery and processes, a high level of professionalism and technical expertise, and independence. Each of these cultural values fed into the regulator's reputation. However, although these cultural values supported the regulatory mission, potential downsides were also identified. An emphasis on delivery and process can lead to unnecessary bureaucracy and overcomplication. Similarly, a strong focus on professionalism can encourage perfectionism, leading to work intensification, and the need for independence can be perceived as arrogance or aloofness, stifling opportunities for cooperation and collaboration.
2.	Although having a strong reputation is an important driver of positive cultural influences, there is also a danger that the emphasis placed on reputation and the need to protect it can lead to negative effects. Both risk aversion and reluctance to challenge emerged as “dark side” effects of protecting the regulator's reputation. As a further consequence, these “dark side” effects can also impact on the regulator's ability to engage in feedback and self‐reflection, hindering learning and innovation, and slowing the pace of change.
3.	The predominant style of leadership was based on consensus, which can be effective in building commitment and support among staff, but can also undermine accountability. Internally, the regulator was perceived as very supportive, particularly within teams. However, this also led to tensions between regulatory and corporate staff, especially where the regulatory function perceived they did not need support from the corporate side (e.g., human resources, finance). From an external perspective, the regulator was seen as open and supportive, and taking a constructive approach with stakeholders.

We report our detailed findings in the sections below (see Table 2 for illustrative quotes), focusing on three key interrelated areas: core values; enacted behaviors and elaborating practices; and enabling factors (Vogus et al., 2010).

**TABLE 2:** Illustrative quotes to support thematic analysis.

Theme	Interview / focus group illustrative quotes
Reputation	“*Reputation is so important, how you conduct yourself, how you manage yourself on site, the impression you give it's absolutely crucial for being a good regulator*” (Focus Group 5) “*If the regulator is perceived as weak then this will have a knock on effect on how dutyholders behave and may have negative safety implications*” (Focus Group 9) “*The way we behave and our culture, influences their [dutyholders’] culture*” (Focus Group 9) “*It was my comment about the avoidance of reputational damage—I think when a priority comes along to make a regulatory sound decision, other things can be overtaken or overlooked, you know. But that's the nature of what we produce, you know. That's the output of our production line, if you like, so that becomes the important thing at times*” (Focus Group 6) “*The regulator's so preoccupied by its reputation. And I understand that it is important that we have a good reputation and we inspire confidence. But we can obsess over it and especially reputation when it comes to what industry thinks of us as well*” (Focus Group 5)
Process orientation	“*The regulator is absolutely excellent, but I think it relies on a lot of checks and balances which it's important to maintain in the organisation*” (Focus Group 8) “*It can mainly end up being overly bureaucratic. We can delay things and we can get things out of proportion and actually build a whole bureaucracy there*” (Interview 8, leader)
Professionalism	*“That deep technical competence is what then feeds up into being an effective regulator*” (Focus Group 7) *“It's delivery culture but it's also professionalism. We will—I'm speaking for myself in some ways, but I always go the extra mile*… *For me, I've always done whatever's been asked of me. That's professionalism. That's just what we do.”* (Focus Group 7) *“It all eventually comes down to workload and how, with a highly dedicated workforce, we all want to do that extra bit more. So there's a personal thing on it too.”* (Focus Group 9) *“We work on the principle that if you can do more you should, simple as that isn't it. So like we say, something fits for 97% of the people, we have that approach to say, well actually, no, we want the extra 3% as well.”* (Focus Group 8)
Independence	“*You need to be able to demonstrate to all of them [external stakeholders] that we are just…. independent, absolutely straight line…. we'll apply the law without fear or favour*” (Focus Group 7) *“When you're in a joint meeting, quite often they see themselves as the primary regulator.(…) they tend to sometimes not always see that other regulators have got an interest*… *we've all got skills and if we can bring all our skills together and do things in a more efficient way and trust each other to do things, or trust each other with sharing of information.”* (Interview 4, external stakeholder) *“And that will save us a lot of work, it will save a lot of cost to the dutyholder and yet we put little barriers in our way to—whether they are sort of protocols or regulator interactions. Where really we should be collaborating in amongst the organisation but also between regulators.”* (Focus Group 8)
Challenge	“*This person is so concerned with their own reputation and conservative decision making, they're not prepared to actually stand up and put their name on something*” (Focus Group 5) “*They'd rather just not try than risk it going wrong*” (Focus Group 3) “*Challenge is welcomed, unless you're challenging something that somebody doesn't want you to challenge*” (Focus Group 2)
Risk aversion	“*No one is willing to make the tough decisions*” (Focus Group 7) “*An environment of constant change with no outcome*” (Focus Group 4) “*From a culture point of view we're so risk averse that we don't actually trust what we write ourselves… it's an absolute nightmare…. We push everything up and up and up because of risk aversion*” (Focus Group 8)
Leadership	“*If we do mess up, we put our hands up and say look actually, do you know, I got this wrong, but people don't*… w*e do shy away from having those conversations sometimes….and it is almost kind of accepted, which isn't right*” (Interview 9, leader) *“I've sometimes felt kind of overall regulatory strategy, I've sometimes found it quite sluggish, trying to establish what our overall direction is and when those decisions will be made”* (Focus Group 6) “*Whereas at the moment we've got no direction and that's what's causing so much issue across the organisation ‘cos there is no direction what's expected*” (Focus Group 4) *“We need to be cautious of the feedback we provide because they're feeling very sensitive to it”* (Focus Group 1) “*Challenge isn't universally welcomed, or people pay lip service to it”* (Focus Group 7) *“we're getting asked to provide feedback and challenge, and we do, and either it doesn't get listened to—and that's not just [support staff], this can be inspectors as well”* (Focus Group 4) *ʻʻWe're not a cohesive leadership team, whether it be the senior leadership or this level* (Focus Group 9) *“The people know their behaviours and they're not necessarily always held to account, which is where I lose the trust”* (Focus Group 5)
Supportiveness	*“Support is valued. I never feel unsupported. Exactly as [participant 2] said, I feel like I have a team around me wherever I go. I always feel I'm supported, I always feel backed, so that's fantastic.”* (Focus Group 7) *“I think, from a senior level, we have a good supportive relationship”* (Interview 10, leader) *“I think your own team is supportive and demonstrates the values in terms of giving feedback and all the rest of it, but obviously I don't think your higher‐uppers unfortunately—I don't think they're demonstrating them.”* (Focus Group 4) *“I think they're very supportive of their staff, you know, very, very supportive of their staff, and where staff have made decisions in meetings, you know, they're very good at supporting them and backing them up on that”*. (Interview 11, leader) *“The majority of people, I think are supportive. And the feel of the place, it's kind of a supportive atmosphere and environment. And, you know, Teams meetings, just remote meetings, people that you've not worked with before, not met, you can go back to them and ask them for help”* (Focus Group 2) *“Many people are approachable and willing to help out”* (Focus Group 8)
Change & innovation	*“We are very, very bad in a lot of contexts at innovating new processes and new ways of doing things because we're so conservative”*. (Focus Group 5) *“We're quite traditional so we've got quite a lot of inertia to change. We've always done it that way, so we're always going to continue.”* (Focus Group 8) “*I think it's only when they get to that absolute point of, I understand all the risk and all the data, I've had it independently assured three or four times, we'll make a decision now as long as everyone's collectively on this. Which is great from a regulatory point of view but when you're making a very simple internal decision about, you know, the simplest thing, it's not an efficient way to go about things*.” (Focus Group 2) “*When it comes to thinking about these bigger issues and thinking outside the box rather than following their narrow technical process, which says, okay well we worked at this standard of protection… I'm not sure that necessarily grasps, you know, the subtleties if you like of where we are at*.” (Interview 8, leader) “*New and innovative—yeah, I don't see much evidence of that*” (Interview 7, external stakeholder)

### 3.1 Core values

We focused on identifying the core values that act as the fundamental drivers of regulator culture through the triangulation process. We identified “reputation” as a basic assumption (i.e., a taken‐for‐granted, largely unconscious, underlying belief), because it provided a common explanatory narrative underpinning patterns of attitudes, beliefs, and behavioral norms. It emerged consistently through the data analysis, and as an overarching theme across data sources (see Figure 2), leading the research team to identify reputation as a basic assumption (see Figure 3). Regulatory reputation emerged as a fundamental assumption, which was underlying other strongly held, consistently shared values: process orientation (i.e., focus on delivery and procedures), professionalism (i.e., focus on technical competence), and independence (i.e., focus on freedom from undue influences). These values align with the cultural characteristics of a “strong regulator” identified in previous research, which reflect perceived credibility, competence, and effectiveness (e.g., Reiman & Norros, 2002).

#### 3.1.1 Reputation

Regulatory staff were highly cognizant of potential effects on the regulator's reputation, and their role in maintaining a strong reputation. The focus on reputation was reflected in the document analysis, as well as how we observed inspectors to conduct themselves onsite. Reputation was highly influential for the regulator's ability to regulate effectively, which relies not only on its legal powers to use enforcement action, but also crucially through encouraging good practice and improvement. The latter rests much more on powers of influence rather than legal authority, which is supported by a strong reputation.

The ability to influence, using a constructive regulatory approach, cannot be achieved without the dutyholder believing in the regulator's credibility and legitimacy. In interviews, dutyholders discussed their appreciation of this regulatory approach, particularly in terms of openness (e.g., “*there are no sensitivities*,” “*we can talk transparently about anything*,” and “*open to challenge*”) and support (“*there is a mutual respect*,” “*engaged*,” “*helpful*,” “*feel listened to*,” and “*trustworthy*”).

In addition, however, participants highlighted the “drive to protect reputation,” which was rooted in the regulator's “*preoccupation with reputation”* and its focus on “*avoiding reputational damage.”* While understanding the need to inspire confidence, regulatory staff described how the drive to protect the regulator's reputation, could affect decision‐making, and make staff anxious about making mistakes.

#### 3.1.2 Process orientation

A clear focus on process orientation was viewed as important for maintaining high standards of performance, with staff describing delivery as “*king”* and *“prioritized*.” Process orientation was evident in the document analysis (e.g., extensive, highly detailed documentation), and was spoken about by external stakeholders *(“they love process, they love procedure*”). One external stakeholder highlighted how the regulator's “excellence” depends primarily on having all their processes and procedures in place.

However, a strong process orientation was associated with the tendency to create too much procedure, structure, and bureaucracy, which led to struggles with simplification. Participants mentioned how process can be prioritized over expertise, hindering the capacity for continuous learning and improvement.

#### 3.1.3 Professionalism

Staff were described by external stakeholders as having highly professional attitudes, being *diligent, hard‐working, task focused, rigorous*, and *perfectionist*. The “*deep technical expertise”* of inspectors was seen to earn respect and give regulatory advice its credibility. This was evident during observed interactions with dutyholders, as well as in discussions with external stakeholders.

Staff highlighted that their commitment as professionals led to a willingness to “*go the extra mile*” and do “*whatever's been asked of me*,” which can lead to work intensification (such as, long work hours and heavy workloads). A focus on excellence and aspiring to high standards emerged as an important element of professionalism. This was reflected in comments from staff and managers that there is a sense that “*nothing is good enough unless it is perfect*.” While this encouraged a high level of staff commitment, it was also perceived as creating an overly “*challenging*” and “*competitive*” environment, which can erode psychological safety (e.g., making staff fearful of admitting mistakes) and stifle the expression of new thinking.

#### 3.1.4 Independence

Independence (i.e., the ability to stand apart and be seen as not unduly influenced by external parties) was perceived as critical to building and maintaining the regulator's reputation. Staff discussed the importance of demonstrating to external stakeholders that they apply their powers “*without fear or favor*.” Independence emerged as a subtheme through the initial data analysis stages (see Figure 2), and was strongly endorsed as a major theme through discussions with external stakeholders, observations, and document analysis, in the final model.

However, maintaining independence had implications for collaboration. This was evident in observations of regulator interactions with dutyholders and other regulatory bodies. Indeed, external stakeholders perceived barriers to collaborative working with the regulator, especially when the regulator perceived itself to be the “*primary regulator*,” without taking sufficient account of others’ perspectives. Participants provided examples of “*technocratic*” decisions, whereas effective collaboration might have allowed for the consideration of wider ramifications.

### 3.2 Enacting behaviors and elaborating practices

“Enacting behaviors” (Vogus et al., 2010) are organizational behaviors that manifest the underlying cultural values. We identified two themes related to enacting behaviors, which reflect how the regulator responds to challenge and manages risk. Themes related to challenge and risk highlight the behavioral consequences of the drive to protect the regulator's reputation. Thus, reputation not only underpinned the regulator's ability to influence, but also generated a need to protect reputation, leading to behaviors characterized by reluctance to challenge and risk aversion.

A third theme we identified as “change and innovation,” which should act as “elaborating practices” (Vogus et al., 2010) that feedback into the regulator culture, allowing for adaptation and growth. This feedback mechanism leads to strengthening of cultural values, but can be maladaptive. We found that this tends to reinforce reputation protection, rather than encouraging learning through constructive challenge and openness.

#### 3.2.1 Reluctance to challenge

This theme included the ability to challenge appropriately, where staff spoke about a “*lack of challenge*,” “*lack of self‐reflection*,” and “*challenge is not always welcome*.”

The need to protect reputation led to staff being fearful of failing (“*Because of the reputational aspect we are not allowed to fail”)*. Given the focus on preventing reputational damage, we found that this affected staff behavior, including the willingness to accept accountability for their actions, and apprehension about actions not going to plan (“*Fear of making mistakes*”; “*mistakes are not forgotten*”).

#### 3.2.2 Risk aversion

Participants (both internal and external) described the regulator culture as “*change‐averse”* and “*cautious*.” Conservative decision‐making was perceived to be a strength and expected within a regulatory context. However, having a “*technocratic mindset”* was characterized by inflexible behaviors, and a strong focus on long‐standing processes. Some interviewees questioned the regulator's ability to “*think outside the box*,” which seemed to hinder its adaptability in the face of new developments.

There was evidence of a risk‐averse approach being transferred into strategic activities, such as enacting change, leading to a slowed pace of progress, especially in terms of implementation. This led to perceptions of “*constant change with no outcome*.”

#### 3.2.3 Change and innovation

A major theme of change and innovation emerged. This reflected strong commitment to the values of process orientation, professionalism, and independence, but could lead to limited opportunity for change and innovation (e.g., by following elaborate decision‐making processes). These behaviors impact upon the regulator's capacity to learn, enact strategic change, and innovate (see Figure 3).

Process orientation affected decision‐making processes, described by staff as being highly formalized, leading to unnecessary bureaucracy. Similarly, professionalism fostered an attitude that “*everything has to be perfect*,” which contributed to protracted processes of decision‐making, slowing down the management of change. Furthermore, a sense of “*not being allowed to fail”* impeded the regulator's capacity for learning and change. In a working environment in which people are fearful of admitting mistakes, errors do not become opportunities for learning. Similarly, an environment that fails to encourage people to speak up can stifle opportunities for learning.

### 3.3 Enabling factors

In our model, we positioned leadership and supportiveness as “enabling factors” of regulator culture (see Figure 3). Leaders create the conditions for the development of cultural values, by directing attention, and empowering employees to speak up (Vogus et al., 2010), while supportiveness, at both organizational and team level, creates the conditions for team effectiveness, and psychological safety within teams, fostering shared cultural understanding (Bisbey et al., 2021).

#### 3.3.1 Leadership

Leaders can enable culture by acting as role models (Bisbey et al., 2021). However, while there was an understanding that leaders needed to act as role models, this did not always happen, with staff noting that leaders did not always “*put their hands up”* and take accountability for their actions.

Our analysis suggested the predominant leadership style within the regulator might best be described as “consensual.” This is a relational form of leadership, which involves facilitating information exchange, encouraging discussion, and resolving dissent within teams (Rowland & Parry, 2009). With an emphasis on gaining agreement, consensual leadership can increase perceptions of collective commitment and provide direction for efforts. However, it was perceived as slow, time‐consuming, and readily derailed through resistance (even by a small minority).

Placatory leadership behaviors were said to affect accountability within the regulator, such as a tendency to avoid difficult conversations. One consequence was a reluctance to deal with negative behaviors for fear of upsetting someone, or rationalize behavior (“*they didn't mean to*”), such that negative behaviors were not addressed.

#### 3.3.2 Supportiveness

The regulator was perceived as supportive at a team level. Staff reported high levels of collegiality and effective team working, with coworkers described as *kind, considerate, respectful*, and *friendly*. In addition, staff felt valued and supported (“*draw on each other's strengths*” and “*gets the best out of people*”). Staff described the regulator as “*a great place to work”* and, although there were critical comments, participants noted that these should be considered in the context of the low turnover rate, and long service of many staff. External stakeholders discussed how the regulator demonstrated support for them. Organizational policies examined in the document analysis fed into this theme.

However, staff also discussed a feeling of “*siloed*” working, and highlighted disconnect across different departments (“*them and us”*). This was reflected in comments about tensions between functions (“*are we valued equally*?”) and how this may act as a barrier to feeling part of a single organization.

## 4 DISCUSSION

Our research was conducted to explore: (1) what is a “regulator culture for safety,” and (2) what cultural values do regulators need for effective oversight of a high‐risk industry? We considered these questions through an abductive case study theory approach, developing a reputation‐focused model of regulator culture (see Figure 3 and Table 1). This model encapsulates the core values of regulator culture, which align with previous research, but extends our understanding of the mechanisms through which these affect the regulator's effectiveness (i.e., through enacting behaviors, and elaborating practices). Here, we highlight the role of reputation as a basic assumption within regulator culture, and how the drive to protect reputation has benefits, but also a potential “dark side.” Furthermore, we emphasize the dynamic and paradoxical nature of cultural values, and how this affects the ability of the regulator to continually improve and strengthen its culture over time, which has implications for effective regulation.

### 4.1 Is a good reputation a dangerous thing?

*“An eminent reputation is as dangerous as a bad one”* (Tacitus).

We identified reputation as central to regulator culture. Reputation may be pertinent for risk regulators, because their power depends on the ability to influence, which in turn, is dependent on their perceived credibility (Maor & Sulitzeanu‐Kenan, 2013), particularly in goal‐based regulatory regimes. It profoundly affects their relationships with external stakeholders, especially how they are perceived by dutyholders (Willis et al., 2024). Furthermore, the drive to protect reputation (i.e., the avoidance of reputational damage or loss), affects organizational behavior (leading to lack of challenge and risk aversion), highlighting its potential “dark side.” This aligns with previous work emphasizing the effects of reputation protection and reputation management tactics (e.g., Carpenter, 2010, 2004; Maor et al., 2013), but has rarely been discussed from a cultural perspective.

Organizational reputation is characterized by fragility, meaning that reputational damage can occur relatively easily, and be difficult to repair (e.g., Ruddle et al., 2023). Thus, organizations may focus on “reputation protection,” and engage tactics to actively manage their reputation to avoid such damage. The implications of reputation protection have emerged in several cases of organizational misconduct, highlighting the potential “dark side” of a good reputation. For example, Phillips (2019) concludes that: “Oxfam GB put protecting its reputation and donor relationships in the short term above protecting those it serves or maintaining public trust over the longer term” (p. 1) in relation to a scandal concerning inappropriate staff behavior. Similarly, NHS investigations have found that Trusts may be more concerned with reputation management, than transparency in dealing with failures. For example, the independent inquiry into maternity services at NHS East Kent (Kirkup, 2022) discussed how organizational behaviors in NHS Trusts “that place reputation management above honesty and openness are both pervasive and extremely damaging to public confidence in health services” (p. 24). Reputation management tactics came to prominence in the case of Lucy Letby, where senior managers at the Countess of Chester Hospital took action to silence, and even sanction, clinicians’ complaints about the nurse's connection to babies’ deaths, rather than investigate them and risk reputational damage (Mathew, 2023).

These examples illustrate how striving to protect a “good reputation” can become a dangerous thing. While actively adopting reputation protection tactics can be damaging, passive effects can also be detrimental. While a regulator would be expected to aspire to excellence, the pressure to protect its reputation can affect accountability, where staff are reluctant to admit to mistakes, and challenge, where staff feel inhibited to speak up. Under such circumstances, this may stifle error reporting, and subsequently affect organizational learning (Lei et al., 2016). Similarly, a regulator would be expected to be risk averse, and make conservative decisions, but this can also affect the ability to implement change and be innovative (Miron‐Spektor et al., 2011). Thus, while a reputation as a strong regulator has distinct benefits, it can also hinder elaborating processes (such as self‐reflection, adaptation, and learning) that act to continuously reinforce culture. This finding aligns with previous work on reputational risk that highlights how regulatory response to reputational threats can increase organizational rigidity, rather than encourage a climate of innovation (Boon et al., 2023). Thus, efforts to protect a good reputation act as an important mechanism linking cultural values to regulatory practices and enhance our understanding of regulator culture.

### 4.2 Paradoxes in regulator culture

Cultural values underpin effective regulatory practice, but there can be “too much of good thing effect” (Pierce & Aguinis, 2013), where strongly held commitment to cultural values has detrimental effects (e.g., bureaucratic, perfectionist, and uncooperative). Furthermore, our findings emphasize the potential for *cultural tensions* (or paradoxes) to develop between core values over time, highlighting the dynamic interplay between factors that is often missing from descriptions of culture (Antonsen, 2017; Hollnagel et al., 2006). Cultural tensions may emerge as antagonistic relationships between specific values within the culture, which are both contradictory and interdependent, well‐embedded, and persistent over time (Lewis et al., 2014). First, process orientation reflects the value placed on processes and procedures, which is necessary within regulation, but can hinder continuous learning and improvement. Second, professionalism reflects the emphasis on technical competence and expertise, which builds credibility as a regulator, but can lead to a competitive working environment that hinders psychological safety and the expression of new thinking. Third, independence of the regulator is critical, but affects the ability to collaborate effectively.

Understanding these paradoxes provides insight into how cultural values interact to affect regulatory practice, and emphasizes the need for regulators to foster cultures that encourage a “paradox mindset” (Miron‐Spektor et al., 2018). In order to address these tensions, a paradox mindset (which recognizes and embraces cultural tensions) is needed throughout the organization, at a micro level (e.g., inspector behaviors), but also at a macro level (e.g., leader actions). At the organizational level, this ties into existing theorizing on the characteristics of high‐reliability organizations (Cantu et al., 2020; Weick, 1987), which includes paradoxical capabilities, such as “simultaneous centralization and decentralization” (in which organizations must utilize both centralized control and decentralized expertise). One implication is that leaders of regulatory bodies should aim to actively manage such tensions, using paradoxical leadership (Zhang et al., 2015), particularly as a means of encouraging innovation (Lee et al., 2023; Zhang et al., 2022). Although less research has focused on individual actions, regulatory staff may also face challenges with embodying values that are seemingly contradictory (e.g., both maintaining independence and collaborating with stakeholders), but research suggests that this can be achieved through a dynamic interplay between values (Putnam et al., 2016), to create a symbiotic relationship (e.g., enhancing perceptions of independence through collaborative interactions with stakeholders).

### 4.3 (Dis)enabling factors of regulator culture

We identified both leadership and supportiveness as enabling factors, which create the conditions for regulator culture to develop (Vogus et al., 2010). These factors build and maintain cultural values, but can also undermine them (e.g., through lack of accountability, empowerment, or sense of unity). Furthermore, these factors demonstrated the importance not only of externally‐facing relationships with stakeholders (e.g., dutyholders), but also internally (e.g., between functions). Our analysis showed how the strong commitment to cultural values and drive to protect reputation could have negative consequences for internal processes and relationships.

Leadership, particularly senior managers’ commitment and actions, has featured consistently in research on regulator safety culture (e.g., Bradley, 2017; Fleming & Bowers, 2016, 2019). Regulatory guidance has similarly emphasized how leadership, particularly at senior level, is critical for effective regulation, for example: “the way in which senior management lead [the regulator]… can affect both its internal culture and morale, and its external credibility” (UK National Audit Office, 2021, p. 17). This guidance highlights the importance of the regulator's internal culture for effective regulation, such that the whole organization (not only staff in regulatory roles) are fully engaged and empowered toward achieving effective regulation. We found that supportiveness played a similar role, both at organizational and team level, by encouraging staff to feel valued and empowered, and so supporting culture development. Safety research has provided ample evidence of the importance of organizational and coworker support for safety (e.g., Clarke, 2010; Griffin & Curcuruto, 2016), but to date, there has been limited insight into how supportiveness (e.g., through teamwork) might contribute to culture development (Salas et al., 2020).

### 4.4 Practical implications

Our research demonstrates the practical value of an independent culture assessment to inform regulators, and other interested parties, about their culture. In particular, our methodology (which combines multiple data sources and methods through triangulation) provides a rich description of regulator culture. It has the advantage of examining basic underlying assumptions and core values, including insights into the cultural dynamics at play. Our methodological approach aligns with best practice guidance for risk regulators (e.g., IAEA, 2016), and similar techniques documented previously in the literature (e.g., Schöbel et al., 2017). In addition, there are practical implications for organizations, in terms of ongoing cultural assessment, as the outcomes can form the basis for self‐reflection, adaptation, and learning at an organizational level. This is particularly important as processes of reflection and learning are critical, in turn, for reinforcing regulator culture (Vogus et al., 2010).

Cultural insights can be utilized by organizations in practice to make improvements, not only in terms of managing culture, but also in how cultural values influence staff attitudes and behaviors. For example, such insights might inform the design and implementation of change initiatives and leadership development (e.g., supporting managers to use paradoxical leadership techniques). Our model also illustrates how culture affects strategic processes, such as building agility and resilience, managing and adapting to change, and facilitating innovation. Understanding the culture is the first step to supporting these processes (e.g., senior managers are better able to engage staff and gain commitment to change initiatives).

## 5 LIMITATIONS AND FUTURE RESEARCH

Our research was conducted to explore regulator culture based on a case study of a UK risk regulator. This approach provided depth and richness to our findings, and allowed unique insights into the nature of regulator culture. Our findings are transferable to other regulatory contexts, taking into account relevant boundary conditions, such as the nature of the regulatory regime. Our multisource methodology is replicable, and we would encourage other researchers to utilize this approach to conduct culture assessments of regulatory bodies and gather additional rich descriptions of regulator culture.

However, despite notable strengths, including methodological rigor and triangulation across multiple data sources and methods, there are also limitations.

Given that we worked alongside the regulator to conduct our culture assessment, it is possible that this affected participants’ willingness to speak up, or provide critical contributions to the research. However, we attempted to mitigate these effects, by ensuring the confidentiality of participants, and emphasizing the voluntary nature of their participation. In all communications, the regulator stressed the importance of having external researchers conduct an independent assessment of the organizational culture, which also helped reassure participants. These mitigations give us confidence that we were able to minimize this limitation, although we acknowledge the potential effects.

In addition, our approach was abductive, aiming to describe culture “as is,” rather than comparing the culture to a predetermined notion of what it “should be.” This means that we cannot provide specific recommendations about what actions might be taken to “improve” culture based on our findings.

Reflecting on the sense‐making processes in which we engaged to develop our model, we initially drew on Schein's (1990) theory, using its terminology to describe basic assumptions and values, which is commonly utilized in safety culture research (Casey et al., 2022). However, as the data analysis progressed, we drew on additional theory, and found Vogus et al.’s (2010) process model helpful in considering how themes mapped onto enabling, enacting, and elaborating actions. Thus, the latter model is transferable outside the healthcare safety context in which it was developed. Future researchers might seek to establish a consistent “language” in how we discuss culture, which has been a contentious issue for researchers over several years (Edwards et al., 2013; Guldenmund, 2000, 2010; Hopkins, 2006). In addition, our findings provided insight into the “black box” of regulator culture, in terms of identifying not only the core values, but also the underlying mechanisms linking regulator culture to effective regulatory practices, as well as how paradoxes manifest as part of the culture. We believe that this is a fruitful line of future enquiry, given the limited attention that such research has attracted.

## 6 CONCLUSIONS

Regulator culture is a valuable construct that deserves renewed attention by risk and safety scientists. Our study identified key cultural values that have both positive (e.g., reflecting the credibility and competence of the regulator) and negative effects (e.g., reducing speaking up and accountability, and increasing risk aversion among regulator staff), and identified the culture as driven by the need to protect regulator reputation. Our work has expanded theoretical frameworks by better understanding the intersection between safety culture and reputational risk, with both perspectives reflected in our model of regulator culture. A deep dive into regulator culture has revealed its complex and multifaceted nature (e.g., the existence of cultural tensions and their effects) and highlights opportunities for further theory development and research (e.g., the value of a holistic approach to regulator culture). There are multiple benefits to a better understanding of regulator culture, including improvements to internal processes at the regulator, as well as their regulatory practices and interactions with dutyholders, leading to more effective regulation in high‐risk industries.

## CONFLICT OF INTEREST STATEMENT

The authors declare no conflicts of interest.

## Supporting information




Supporting Information

## Data Availability

The data that support the findings of this study are available on request from the corresponding author. The data are not publicly available due to privacy or ethical restrictions.

## Footnotes

1 In the United Kingdom, effective regulatory practices are defined within a goal‐based regulatory context, as “taking an inclusive and collaborative approach, being proportionate and adaptable, [and] being outcomes‐focused and future‐facing” (Better Regulation Framework, UK Government, 2023, p. 38). These are relatively high‐level outcomes of implementing regulatory principles and policies.

2 The data are drawn from a larger data set that includes quantitative survey data, but we only utilized the qualitative data collected in the current analysis.
