# Supplementary material for: Is a good reputation a dangerous thing? A multimethod assessment of regulator culture and the implications for risk regulation
Source: Risk Anal. 2025 Apr 24;45(9):2650–66. doi: 10.1111/risa.70043 (PMC12474531; doi:10.1111/risa.70043)
Supplement: Supplementary file 1 — Supporting Information [file RISA-45-2650-s001.docx]

**APPENDICES to be included as supplementary materials**

**APPENDIX A:** Topic Guide for Focus Groups

Describe the culture of the regulator (using Ketso exercise)

We are doing these Focus Groups to better understand the culture of the regulator. What we mean by culture in general terms is ‘how things are done around here’, - so we are not only interested in “what” kind of things you do at the regulator but also in “how” things get done. What is valued and rewarded here and how does that influence the way work is done? With this in mind:

1. Can you write down what the culture at the regulator is for you? - What are some of the key things that would describe and influence “how things are done around here”? - (I will put up instructions on a slide)

**once all participants have written down their answers, we will ask each individual to read out one leaf at the time and ask them to put it on the felt (all branches will be blank at that point).*

- 1. Why? *(Explain what you wrote on your leaf – what does it mean?)*

**If anyone has written the same (or something very similar) – they can add to the conversation and then put their leaf on the felt next to it (Clusters may form here) – then we move on to the next person to share their leaf.*

*
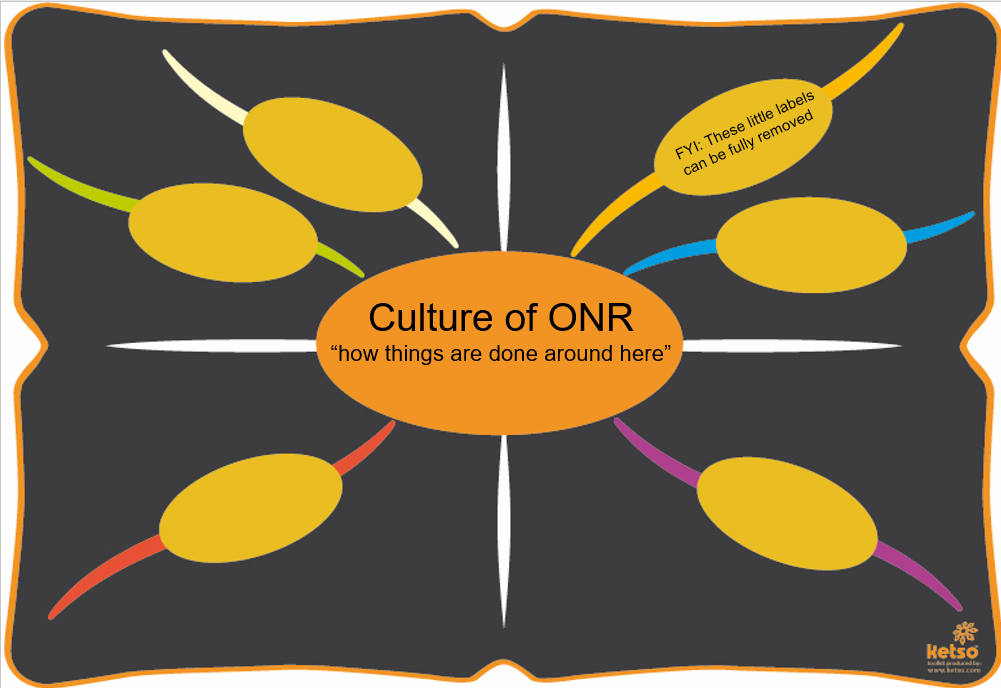
*

- 1. Where does it fit?

**Once we have gone through all the leaves and clustered them on the empty felt, I will ask participants if the kind of things they wrote down fit under those dimensions and if so, why they see them there – they can then move the leaves to the relevant labelled branches.*

- *If they do not fit anywhere there are blank ones for new themes.*
- *If the group has not touched on certain dimensions (e.g., no leaves have been placed there), I will ask them about the dimension before moving on to step 2.*

Example felt with labels:


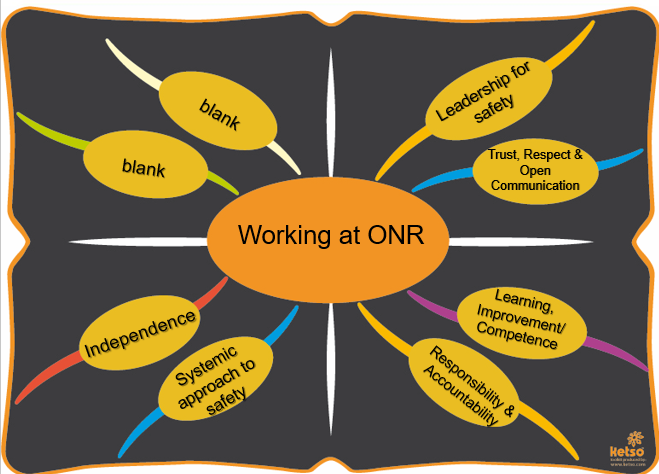


* End of Ketso exercise and start of general discussion*

1. Do you think people would agree with this in other parts of the organisations?

*(Different ways to phrase the question: To what extent do you think people in other departments would agree with what we talked about? / Do you think there are differences between your department and the rest of the regulator? / Do you think there are any other differences within the organisation in how culture might be perceived?)*

1. What happens if things don't go as they are planned/are not implemented well?

Prompts/follow up: If something serious happens - what happens then?

Do you think that is the same in the organisation?

**APPENDIX B:** Questions for semi-structured interviews

**Internal stakeholder**

We are doing these interviews to better understand the culture of the regulator. What we mean by culture in general terms is ‘how things are done around here’, - so we are not only interested in “what” kind of things you do at the regulator but also in “how” things get done. What is valued and rewarded here and how does that influence the way work is done?

- How would you describe the culture at the regulator?
  - What words would you use to describe the culture?
  - How does it feel to work here, versus other places that you have worked?
  - Is there anything ‘special’ about the regulator that makes it the workplace that it is?
  - Are there any characteristics that you need to be successful at the regulator?
  - Is there anything about the culture that makes you feel uncomfortable?
- Do you think there are differences in culture in different parts of the organisation?
  - Are things done differently?
  - Do some sections have a different ‘feel’ as a place to work, or to interact with?
  - Do you find it difficult to engage with people in different parts of the organisation?
  - To what extent do you think that people at the regulator share a common ‘language’?
- How do you think that the regulator’s culture supports its mission?
  - To what extent do you see the regulator’s values ‘lived’ on a daily basis – can you give me some examples?
  - What about when something goes wrong, or something unexpected happens, how does the regulator react? Any examples?
  - Does the regulator have any particular ‘sensitivities’ – things that are difficult to discuss openly, or raise with leaders?
  - Do you think that there is anything that holds the organisation back in achieving its mission?
  - What do you think should be a priority for change? If so, could you say why?
- How do you think that the leadership at the regulator affects its culture?
  - To what extent do you think that leaders in the regulator ‘walk the talk’?
  - In different functional areas / sections of the organisation?
  - What do you think that leaders at the regulator do particularly well? Any examples?
  - Is there anything that leaders at the regulator do or say, that you think could be done in a better or more effective way?
- Are there any aspects of culture at the regulator which we haven’t discussed that you believe are important?
  - Do you have any further thoughts about the culture and how the regulator achieves its mission as a regulator that you think would help us?

**External stakeholder**

We are doing these interviews to better understand the culture of the regulator, and how it achieves its mission as a regulator. What we mean by culture in general terms is ‘how things are done around here’, - so we are not only interested in “what” kind of things that the regulator does, but also in “how” things get done. What is valued, and rewarded, and how it interacts with licence-holders / government departments / its external stakeholders (including the public)?

- How would you describe the culture at the regulator?
  - What words would you use to describe the culture?
  - How does it feel to interact with the regulator?
  - Is there anything ‘special’ about the regulator that makes it effective / not effective as a regulator?
  - What is it like to deal with the regulator?
  - Is there anything about dealing with the regulator that makes you feel uncomfortable?
- How do you think that the regulator’s culture supports its mission?
  - To what extent do you see the regulator’s values ‘lived’ in their interactions with you – can you give me some examples?
  - What about when something goes wrong, or something unexpected happens, how does the regulator react? Any examples?
  - Does the regulator have any particular ‘sensitivities’ – things that are difficult to discuss openly, or raise with them?
  - Do you think that there is anything that holds the organisation back in achieving its mission?
  - What do you think should be a priority for change? If so, could you say why?
- How do you think that the leadership at the regulator affects its culture?
  - To what extent do you think that leaders in the regulator ‘walk the talk’?
  - What do you think that leaders at the regulator do particularly well? Any examples?
  - Is there anything that leaders at the regulator do or say, that you think could be done in a better or more effective way?
- Are there any aspects of culture at the regulator which we haven’t discussed that you believe are important?
  - Do you have any further thoughts about the culture and how the regulator achieves its mission as a regulator that you think would help us?

**APPENDIX C:** Topic Guide for Observations

**Regulator Inspections**

The researchers will conduct naturalistic observations, where inspectors and duty-holders are observed in their normal settings and activities without any manipulation or interference by the observers. Observations include notes on the following:

- Body language / non-verbal communication during interactions
- Patterns of interaction / prevalence of interactions with the different personnel (e.g., interactions with leaders vs other staff)
- Information flow between the parties / contributions by different personnel
- Personal space / level of (in)formality
- What was said in conversations between inspector and duty-holder staff
- How duty-holder staff act and react to the inspector (& vice versa)
- Level of engagement / type of questions asked
- Subjective reactions (e.g., the ‘feel’ of the discussion, sense of the atmosphere, etc)

**Regulator Meetings**

The researchers will conduct naturalistic observations, by joining virtual meetings as a passive observer (without any manipulation or interference of the meeting). The researcher will not participate in the meeting, but will take field notes on their observations during the meeting. The meetings will not be audio or video recorded. Questions and prompts to guide the observer in their note-taking include:

- Is there evidence of constructive challenge and questioning (e.g. peer to peer and between levels of seniority)?
- Are clear decisions made and responsibilities assigned for any follow-up actions?
- Is relevant learning (internal, external, nuclear and other industries) discussed and actively used during the discussions?
- Do managers and meeting chairs encourage challenge and questioning, respect others and listen to those present?
- Are any issues and concerns raised?
- Do managers and meeting chairs respond in a positive way and take appropriate action?
- Are participants’ contributions recognized and praised?
